# Benchmark Dose for Urinary Cadmium based on a Marker of Renal Dysfunction: A Meta-Analysis

**DOI:** 10.1371/journal.pone.0126680

**Published:** 2015-05-13

**Authors:** Hae Dong Woo, Weihsueh A. Chiu, Seongil Jo, Jeongseon Kim

**Affiliations:** 1 Molecular Epidemiology Branch, National Cancer Center, Goyang-si, Korea; 2 National Center for Environmental Assessment, U.S. Environmental Protection Agency (EPA), Washington, DC, United States of America; University of Texas Health Science Center at Houston, UNITED STATES

## Abstract

**Background:**

Low doses of cadmium can cause adverse health effects. Benchmark dose (BMD) and the one-sided 95% lower confidence limit of BMD (BMDL) to derive points of departure for urinary cadmium exposure have been estimated in several previous studies, but the methods to derive BMD and the estimated BMDs differ.

**Objectives:**

We aimed to find the associated factors that affect BMD calculation in the general population, and to estimate the summary BMD for urinary cadmium using reported BMDs.

**Methods:**

A meta-regression was performed and the pooled BMD/BMDL was estimated using studies reporting a BMD and BMDL, weighted by sample size, that were calculated from individual data based on markers of renal dysfunction.

**Results:**

BMDs were highly heterogeneous across studies. Meta-regression analysis showed that a significant predictor of BMD was the cut-off point which denotes an abnormal level. Using the 95th percentile as a cut off, BMD5/BMDL5 estimates for 5% benchmark responses (BMR) of β2-microglobulinuria (β2-MG) estimated was 6.18/4.88 μg/g creatinine in conventional quantal analysis and 3.56/3.13 μg/g creatinine in the hybrid approach, and BMD5/BMDL5 estimates for 5% BMR of N-acetyl-β-d-glucosaminidase (NAG) was 10.31/7.61 μg/g creatinine in quantal analysis and 3.21/2.24 g/g creatinine in the hybrid approach. However, the meta-regression showed that BMD and BMDL were significantly associated with the cut-off point, but BMD calculation method did not significantly affect the results. The urinary cadmium BMDL5 of β2-MG was 1.9 μg/g creatinine in the lowest cut-off point group.

**Conclusion:**

The BMD was significantly associated with the cut-off point defining the abnormal level of renal dysfunction markers.

## Introduction

Cadmium is a naturally occurring element that is widely dispersed in the environment at low concentrations. It is often emitted from industrial activities, such as metal mining and refining, which results in occupational exposure to cadmium [1]. The lungs are the target organs for cadmium in occupationally exposed workers because cadmium enters body through inhalation, which can result in an acute high dose. Cadmium has been classified as a human carcinogen [2]. In the general population, the majority of the cadmium exposure in the human body occurs by the intake of cadmium-containing food such as fish and shellfish, except in the case of smokers [3]. It has been estimated that the amount of cadmium per cigarette is about 1–2 μg and about 10% is inhaled when a cigarette is smoked [4]. However, smoking is often considered as a confounding variable in the assessment of cadmium exposure due to the large number of toxic compounds present [1].

Cadmium is less efficiently absorbed from the gastrointestinal tract than in the lungs. The absorption rate of cadmium in the gastrointestinal tract in humans has been estimated at approximately 3–5% [5] and can increase when the iron stores as assessed by serum ferritin are low [6]. Ingested cadmium accumulates mostly in the liver and kidney; however, the critical target organ for chronic low dose exposure is the kidney [1,7]. Although cadmium exposure in the general population is relatively low, cadmium has a long biological half-life in the body and a slow excretion rate. The estimated half-life in humans is 4–19 years in the liver tissue and 6–38 years in the kidney [1]. Chronic oral intakes of low dose cadmium can lead to cadmium accumulation in the kidney. The metallothionein-cadmium complexes prevent kidney damage, but cadmium become very toxic when the kidney cannot produce enough metallothionein. Chronic exposure to a low level of cadmium is associated with renal tubular dysfunction [8]. Many studies have shown a dose-response relationship between urinary cadmium and renal dysfunction markers, such as low molecular weight proteins [α1-microglobulin (also referred to as protein HC), β2-microglobulin (β2-MG), retinol binding protein (RBP)], intracellular tubular enzyme [N-acetyl-β-d-glucosaminidase (NAG)], and high molecular weight proteins [albumin (ALB)] [9].

To set a reference value for cadmium exposure, the levels of exposure that do not cause appreciable adverse health effects should be estimated. The no-observed-adverse-effect level (NOAEL) approach was traditionally used to set a point of departure for a guideline value of the human exposure limit. Later, the benchmark dose (BMD) was introduced for the risk assessment of contaminants [10,11]. The BMD is the dose level causing a specified change in the response (referred as the benchmark response, BMR) of a health effect, which is derived from the dose-response curve fitted to data. The BMD is generally defined as corresponding to an extra or additional risk of 5 or 10% of the BMR, although the methods to derive BMD and the one-sided 95% lower confidence limit of benchmark dose (BMDL) are varied. The BMD is less dependent on dose spacing and make full use of all dose-response data compared with NOAEL [12,13]. Thus the BMDL has been proposed as an alternative to the NOAEL [14].

Urinary cadmium, which is sensitive biomarker of long-term exposure, has been used as an index of the total body burden, and renal dysfunction has been used as a marker for adverse health effects [6]. Studies have reported the BMD/BMDL derived from urinary cadmium and renal dysfunction markers of a population from cadmium polluted or non-polluted area. Another approach is to conduct meta-analyses to determine the BMDs/BMDLs from a dose-response relationship between urinary cadmium and β2-MG using geometric means and standard deviations observed in each of the reported studies [3,15].

We conducted a meta-analysis using reported BMDs and their corresponding standard errors that were calculated from individual data, and we attempted to compare the results of previous reports as well as to find the factors that affect the BMD in the general population.

## Material and Methods

### Study selection

A systematic search for relevant studies written in English was conducted with PubMed using the terms (benchmark dose) and (cadmium) up to April 30, 2013. A manual search with a reference list of selected journals was also performed. The inclusion/exclusion criteria were as follows: (1) the article reported the cadmium BMD that was calculated using urinary cadmium concentration and markers of renal damage; (2) the article did not derive BMDs using life-time or rice cadmium intake; and (3) the article reported the cadmium BMD and BMDL.

### Data collection

Data on the authors, publication year, country in which the study was performed, environmental cadmium exposure (exposure type), cut-off points for β2-MG (μg/g creatinine) and NAG (U/g creatinine), concentration of β2-MG (geometric mean) and NAG (geometric mean), urinary cadmium concentration (geometric mean, μg/g creatinine), sample size, and BMD and BMDL were collected for the meta-analysis. If only the mean concentrations of each group were reported, the overall mean concentration was calculated based on the number of samples in each group and their mean concentrations for the meta-regression analysis [16–19].

### Statistical analysis

All statistical analyses were performed using the STATA software package (version 12, Stata Corporation, College Station, TX). Summary estimates of BMD_*i*_ and BMDL_*i*_ were calculated by weighting sample size (n_*i*_) of the study [20], *i*.*e*., summary BMDL = Σ(n_*i*_×BMDL_*i*_)/Σn_*i*_. The heterogeneity across studies was tested using the I^2^ test according to Higgins et al. [21]. A meta-regression was performed to identify both the cause of the heterogeneity and the associated factors. The urinary cadmium level, the cut-off point (as a continuous variable), BMD calculation method (hybrid vs. quantal model), and sex (total and male vs. female subjects) were used for BMDL5 and BMDL10 in the meta-regression.

## Results

### Characteristics of selected studies

**Fig 1:**
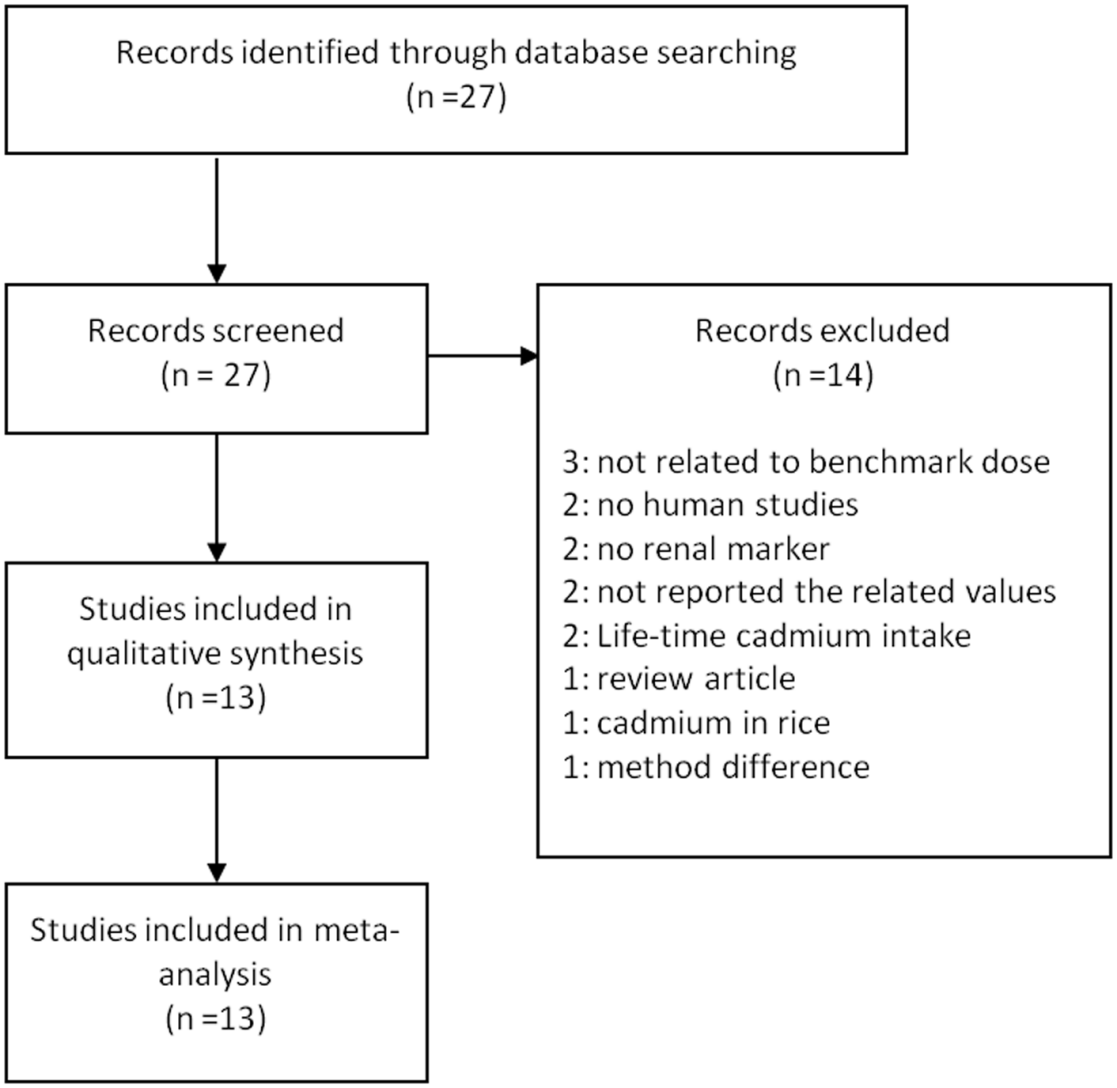
Flow diagram of the study selection process.

A total of 27 studies were identified and 14 studies were excluded due to the following reasons: 3 studies did not include BMD; 2 studies did not include human population; 2 studies did not include renal damage markers; 3 studies included BMD for lifetime cadmium intake or cadmium concentration in rice; 3 studies did not include cut-off points or calculation method for BMD; and 1 study was a review article. Thirteen studies were finally selected for the review of the BMD derived from cadmium-induced renal damage (Fig 1) [16–19,22–30]. The most studied renal tubular damage markers were β2-MG and NAG for the urinary cadmium BMD. The BMD calculated from occupationally exposed population were included only in subgroup analysis. Nine studies (21 data points) for β2-MG and 8 studies (16 data points) for NAG were used for the pooled BMD estimates with exception of studies used occupationally exposed population. Selected studies reporting the BMD for the induction of β2-MG and NAG by cadmium were reported (Tables 1 and 2).

**Table 1:** Studies reporting benchmark dose for cadmium-induced β2-MG.

Author (Year)	Exposure type	Population	Model	Cut-off %	Sex (group)	*n*	Cut-off point	BMD5	BMDL5	BMD10	BMDL10
Suwazono (2011)[27]	Non-polluted	Cadmium non-polluted area (Kobayasi, 2006a), > age 50, Japan	Hybrid	95th	M	520	708	3.4	2.6		
				95th	F	700	415	1.7	1.4		
Suwazono (2011)[28]	Non-polluted	INTERMAP(China, US, Japan, UK) subcohort, aged 50 to 59, Japan	Hybrid	95th	M	201	322.1	1.8	1.2		
				95th	F	203	296.7	2.4	1.8		
Suwazono (2011[29])	Mixed	Cadmium polluted (Shimizu, 2006), non-polluted area (Shimizu, 2006, Kobayasi, 2006b), > age 50, Japan	Hybrid	95th	M	2047	915.5	4	3.5		
				95th	F	2565	897.1	4	3.7		
Lei (2007)[24]	Occupational	Smelter workers (M 85, F 18), control (M 29, F 7), China	Logistic	90th	All	139	162.6			5.2	3.8
Shao (2007)[25]	Occupational	A: Cadmium smelting factory, B: Zinc product factory, China	Logistic	95%	All (A)	196	360			4.89	3.63
				95%	All (B)	206	500			5.07	4.2
Chen (2006)[22]	Occupational	Smelter workers (85), control (29), China	Logistic	90%	M	114	187.6			4.58	3.37
Kobayasi (2006)[18]	Non-polluted	Non-polluted area, > age 50, Japan	Log-logistic	84th	M	1114	507	2.9	2.4	5	4
				84th	F	1664	400	3.8	3.3	6.6	5.5
				97.5th	M	1114	994	6.4	4.5	10.2	7.1
				97.5th	F	1664	784	8.7	7.3	12	9.9
Kobayasi (2006)[23]	Non-polluted	Non-polluted area, > age 50, Japan	Log-logistic	84th	M	547	507	2.6	2	4.6	3.6
				84th	F	723	400	1.9	1.6	3.3	2.8
Shimazu (2006)[19]	Mixed	Kakehashi river basin (>50 yrs), 1981–1982, Japan	Log-logistic	84th	M	1527	507	3.7	2.9	5.1	4.2
				84th	F	1865	400	2.6	1.5	4.2	2.7
				97.5th	M	1527	994	4.8	3.9	6.3	5.5
				97.5th	F	1865	784	4.4	3.2	6.4	5.1
Uno (2005)[30]	Non-polluted	INTERMAP subcohort, 1997–1998, Japan	Quantal linear	84th	M	410	233	0.5	0.4	1	0.7
				84th	F	418	274	0.9	0.7	1.8	1.3
Hong (2004)[17]	Mixed	Arsenic-polluted dominating (122) and control (123) area, China	Probit	95%	All	245	300			1.36	1.13
Jin (2004)[16]	Mixed	Cadmium exposed areas (moderately 243, heavily 294), control area (253), China	Logistic	95%	F	488	800	9.98	8.47		
				95%	M	302	800	5.86	4.74		
				95%	All	790	800	8.36	7.31		

μg/g creatinine: Urinary Cd, cut-off point (β2-MG), BMD5, BMDL5, BMD10, BMDL10

84th, 95th, 97.5th: the 84th, 95th, or 97.5th percentile of the β2-MG distribution at background urinary cadmium concentrations

90%, 95%: not reported whether one-sided or two-sided confidence upper limits.

Mixed = Mixed population from polluted and non-polluted area.

**Table 2:** Studies reporting benchmark dose for cadmium-induced NAG.

Author (Year)	Exposure type	Population	Model	Cut-off %	Sex (group)	*n*	Cut-off	BMD5	BMDL5	BMD10	BMDL10
Suwazono (2011)[27]	Non-polluted	Cadmium non-polluted area (Kobayasi, 2006a), > age 50, Japan	Hybrid	95th	M	547	10.7	6.3	4.1		
				95th	F	723	11.1	4.3	3.1		
Suwazono (2011)[28]	Non-polluted	INTERMAP(China, US, Japan, UK) subcohort, aged 50 to 59, Japan	Hybrid	95th	M	201	2.5	1	0.8		
				95th	F	203	3.3	3.2	2.3		
Lei (2007)[24]	Occupational	Smelter workers (M 85, F 18), control (M 29, F 7), China	Logistic	90th	All	139	10.72			5.5	4.1
Shao (2007)[25]	Occupational	A: Cadmium smelting factory, B: Zinc product factory, China	Logistic	95%	All (A)	196	9.4			2.92	2.13
				95%	All (B)	206	12			3.18	2.58
Chen (2006)[22]	Occupational	Smelter workers (85), control (29), China	Logistic	90%	M	114	9.8			3.61	2.72
Kobayasi (2006)[18]	Non-polluted	Non-polluted area, > age 50, Japan	Log-logistic	84th	M	1114	8.2	4.8	3.3	8.3	5.7
				84th	F	1664	8.5	4.7	3.7	8.3	6.4
				97.5th	M	1114	16	12	7.7	16.4	10.3
				97.5th	F	1664	16.6	10.8	8.5	14.8	11.4
Kobayasi (2006)[23]	Non-polluted	Non-polluted area, > age 50, Japan	Log-logistic	84th	M	547	8.2	3.6	2.5	4.4	3.6
				84th	F	723	8.5	3.1	2.2	5.6	4
Suwazono (2006)[26]	Non-polluted	WHILA study, Sweden	Hybrid	95th	F	790	3.6	0.64	0.5	1.08	0.83
Uno (2005)[30]	Non-polluted	INTERMAP subcohort, 1997–1998, Japan	Quantal linear (M)/ log-logistic(F)	84th	M	410	2.4	0.3	0.3	0.7	0.6
				84th	F	418	2.5	0.8	0.6	1.6	1.2
Hong (2004)[17]	Mixed	Arsenic-polluted dominating (122) and control (123) area, China	Probit	95%	All	245	23			1.48	1.24
Jin (2004)[16]	Mixed	Cadmium exposed areas (moderately 243, heavily 294), control area (253), China	Logistic	95%	F	488	15	6.36	5.46		
				95%	M	302	15	7.74	5.83		
				95%	All	790	15	6.7	5.87		

μg/g creatinine: Urinary Cd, BMD5, BMDL5, BMD10, BMDL10

U/g creatinine: cut-off point (NAG)

84th, 95th, 97.5th: the 84th, 95th, or 97.5th percentile of the β2-MG distribution at background urinary cadmium concentrations

90%, 95%: not reported whether one-sided or two-sided confidence upper limits.

Mixed = Mixed population from polluted and non-polluted area

The BMDs were calculated with either 5 or 10% as the BMR level, and the cut-off points for adverse response were defined as corresponding to 84th percentile, 95th percentile, or 97.5th percentile in the control (mainly) or target population. The BMDs were calculated with various models using BMDS software developed by EPA in earlier studies [16–19,22–25,30], but hybrid approach which allows for calculation of BMD directly from continuous data was applied in more recent studies [26–29]. The studies were mostly conducted in Asian populations (China and Japan). The reported or estimated mean urinary cadmium of the studies ranged from 0.8 to 3.49 μg/g creatinine in a non-polluted general population from Japan, from 2.72 to 2.76 μg/g creatinine in occupationally exposed workers from China, and from 1.51 to 8.0 μg/g creatinine in a cadmium polluted/non-polluted mixed area. The BMDs of 5% BMR (BMD5) for urinary cadmium based on β2-MG ranged from 0.5 to 8.7 μg/g creatinine in a non-polluted general population, and from 2.6 to 9.98 μg/g creatinine in a cadmium polluted/non-polluted mixed area. The BMDs of 10% BMR (BMD10s) for urinary cadmium based on β2-MG ranged from 1 to 12 μg/g creatinine in a non-polluted general population, from 4.58 to 5.2 μg/g creatinine for occupationally exposed workers, and from 4.2 to 9.4 μg/g creatinine in a cadmium polluted/non-polluted mixed area. The BMD5s and BMD10s for urinary cadmium based on NAG ranged from 0.3–12 μg/g creatinine and from 0.7 to 16.4 μg/g creatinine, respectively.

### Meta-regression analysis for BMD5 and BMD10

**Fig 2:**
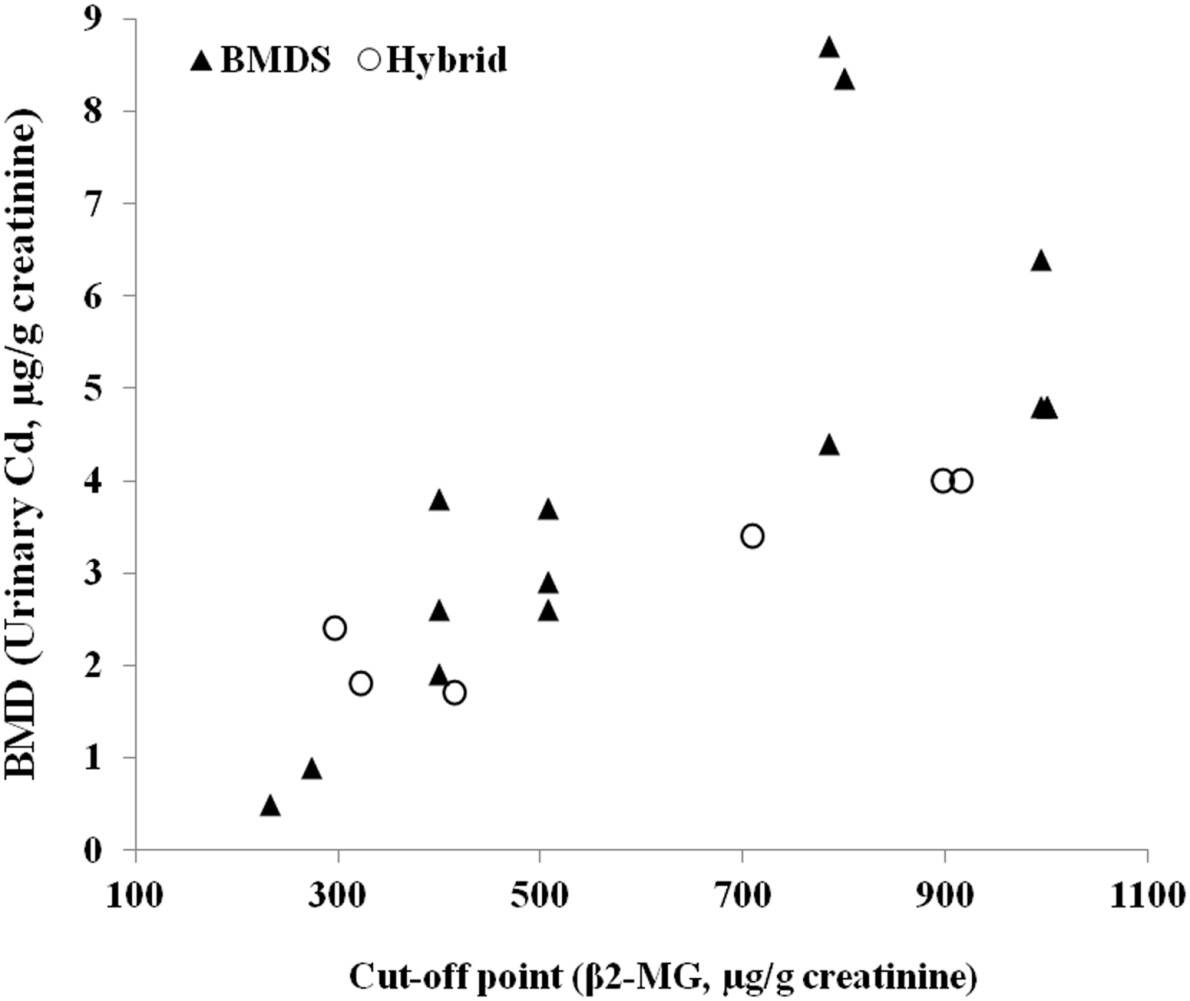
The relationship between cut-off point for adverse effect and urinary BMD for β2-MG. BMDS (▲): BMD calculated by EPA BMDS software, Hybrid (○): BMD calculated by hybrid approach. The positive relationships between cut-off point of β2-MG and BMD in both conventional quantal analysis and the hybrid approach were observed.

Meta-regression analyses were conducted to identify the cause of the heterogeneity across the studies (Table 3). The urinary cadmium level, the cut-off point (as a continuous variable), BMD calculation method (hybrid vs. quantal model), and sex (total and male subjects vs. female subjects) were used in the meta-regression. The meta-regression for BMD of β2-MG showed that the BMD5 and BMD10 were significantly associated with the cut-off point, but BMD calculation method did not significantly affect the results. BMDs for β2-MG were mostly calculated using log-logistic model among conventional quantal analysis. BMD model (probit, quantal linear, logistic vs. log-logistic) was added in the meta-regression, but only cut-off point was significantly associated with BMD for β2-MG (data not shown). The obvious relationships between cut-off point of β2-MG and BMD in both conventional quantal analysis and the hybrid approach were observed (Fig 2).

**Table 3:** Meta-regression analysis for BMDL5 and BMDL10 derived from urinary cadmium and β2-MG.

		β	SE	t	p
BMDL5 (*n* = 14)	Urinary cadmium	-0.238	0.249	-0.950	0.365
	Cut-off point	0.006	0.002	3.830	0.004
	hybrid (vs. BMDS)	-0.588	0.651	-0.900	0.390
	Other ^a^ (vs. women)	-1.229	0.749	-1.640	0.135
BMDL10 (*n* = 9)	Urinary cadmium	-0.503	0.351	-1.430	0.211
	Cut-off point	0.011	0.003	4.370	0.007
	Other ^a^ (vs. women)	-2.372	1.193	-1.990	0.103

BMD from occupational exposure were excluded in the analysis

^a^ Men and both sexes

### Pooled estimates of BMD/BMDL

The pooled estimates for the urinary cadmium BMD/BMDL derived from β2-MG and NAG are presented in Table 4. The meta-analyses were conducted separately by BMR % (BMD5 or BMD10) and cut-off (84th or 95th), and further analyses were performed based on BMD calculation method and sex. The BMD/BMDL varied across the studies, resulting in the between-study heterogeneity being significantly high in most of the groups analyzed except for the occupationally exposed group and 95^th^ percentile cut-off group. In conventional quantal analysis with the 95^th^ percentile as a cut off, the pooled BMD5/BMDL5 estimates of β2-MG were 6.18/4.88 μg/g creatinine and 8.30/6.64 μg/g creatinine, and those of NAG were 8.30/6.64 μg/g creatinine and 12.42/8.86 μg/g creatinine. In the hybrid approach with the 95^th^ percentile as a cut off, the pooled BMD5/BMDL5 estimates of β2-MG and NAG were 3.56/3.13 μg/g creatinine and 3.21/2.24 μg/g creatinine. The pooled estimates were calculated according to the strata of the cut-off points of adverse effects. The urinary cadmium BMDL5 of β2-MG was 1.9 μg/g creatinine in the lowest cut-off point group. BMDL5 increased as the cut-off point increased, but it decreased in the highest strata. The pooled estimates were calculated with the studies that reported for both BMDs of β2-MG and NAG in a study, but no significant difference was observed between the two renal markers in both dose groups (Table 5).

**Table 4:** Pooled estimates of BMD derived from urinary cadmium and β2-MG and NAG.

			Summary	I^2^ (%)^b^		Summary	I^2^ (%)^b^
		*n* ^a^	BMD5/BMDL5	BMD5/BMDL5	*n* ^a^	BMD10/BMDL10	BMD10/BMDL10
β2-MG, Quantal data (BMDS^c^)	Total, 95%	6	6.18 / 4.88	0.0% / 0.0%	5	8.30 / 6.64	84.8% / 83.3%
	Men, 95%	3	5.94 / 4.60	0.0% / 0.0%	2	7.95 / 6.18	0.0% / 0.0%
	Women, 95%	3	6.36 / 5.09	0.0% / 0.0%	2	9.04 / 7.36	0.0% / 0.0%
	Occupational, 95%	-	-	-	4	4.96 / 3.80	0.0% / 0.0%
	Total, 84%	8	2.83 / 2.19	73.8% / 72.1%	8	4.62 / 3.61	55.9% / 66.4%
	Men, 84%	4	2.92 / 2.32	87.2% / 87.0%	4	4.53 / 3.65	75.9% / 83.1%
	Women, 84%	4	2.77 / 2.09	34.6% / 30.2%	4	4.70 / 3.59	0.0% / 11.9%
β2-MG, Hybrid	Total, 95%	6	3.56 / 3.13	0.0% / 0.0%	-	-	-
	Men, 95%	3	3.72 / 3.16	0.0% / 26.5%	-	-	-
	Women, 95%	3	3.43 / 3.11	0.0% / 2.7%	-	-	-
β2-MG, Cut-off point (μg/g creatinine)	162.6–400	7	2.55 / 1.92	70.8% / 66.7%	-	-	-
	407–507	4	2.95 / 2.35	0.0% / 0.0%	-	-	-
	708–800	5	6.25 / 5.02	0.0% / 0.0%	-	-	-
	897.1–994	4	4.54 / 3.81	0.0% / 0.0%	-	-	-
NAG, Quantal data (BMDS^c^)	Cut-off, 95%	2	10.31 / 7.61	0.0% / 0.0%	5	12.42 / 8.86	92.6% / 90.0%
	Occupational, 95%	2	-	-	2	3.67 / 2.79	0.0% / 0.0%
	Cut-off, 84%	5	3.66 / 2.70	96.4% / 93.5%	6	6.25 / 4.64	93.0% / 90.7%
NAG, Hybrid	Cut-off, 95%	5	3.21 / 2.24	81.2% / 74.3%	-	-	-

BMD from occupational exposure were excluded in all analysis, otherwise stated.

^a^ The number of selected data points

^b^ Heterogeneity test

^c^ EPA BMDS software

**Table 5:** Pooled estimates of BMD using studies that reported for both BMDs of β2-MG and NAG in a study.

			β2-MG			NAG		
		*n* ^*a*^	BMD/BMDL	I^2^ (%)^b^	Cut-off	BMD/BMDL	I^2^ (%)^b^	Cut-off
BMR, 5%	Total	13	3.24 / 2.84	0.0% / 16.8%		4.37 / 3.53	63.4% / 67.7%	
	uCd, < 2	7	2.40 / 2.22	0.0% / 0.0%	437.7	3.31 / 2.82	61.2% / 70.4%	7.3
	uCd, ≥ 2	6	5.84 / 4.81	0.0% / 0.0%	630.3	7.70 / 5.75	0.0% / 0.0%	11.3
BMR, 10%	Total	8	7.19 / 5.72	76.3% / 76.5%		9.81 / 7.09	88.4% / 85.5%	
	uCd, < 2	4	3.10 / 2.49	0.0% / 0.0%	370.3	3.87 / 2.93	36.7% / 25.9%	10.6
	uCd, ≥ 2	4	8.62 / 6.84	0.0% / 0.0%	671.3	11.87 / 8.54	0.0% / 0.0%	12.3

^a^ The number of selected data points

^b^ Heterogeneity test

## Discussion

The meta-regression showed that the BMDL was significantly related to the cut-off point for β2-MG, but was not significantly associated with BMD calculation method. The meta-analyses for the BMD/BMDL estimates were conducted using studies that reported BMD and BMDL calculated with urinary cadmium and markers of renal dysfunction. The mean urinary cadmium concentration as well as the BMD of the selected studies varied, and between-study heterogeneity was high in most subgroup meta-analysis.

The studies were mostly conducted in China and Japan. In Japan, a general population aged over 50 years old from either cadmium-polluted or non-cadmium polluted area were included. The study populations were mostly from one of the most cadmium-polluted areas, the Kakehashi river basin. A dose-response relationship between urinary cadmium and renal markers was also shown in the populations from the non-cadmium polluted areas, and the concentration of urinary cadmium varied. In China, the BMDs were calculated using occupationally exposed workers or the general population living in a cadmium polluted area. Studies with occupationally exposed groups showed homogenous results in the summary estimates of the present study. This may be because similar methods for sample collection and bioassay, BMR %, cut-off, and the use of models to estimate the BMD value were used in studies with occupationally exposed workers.

The relationship between cut-off point of β2-MG and BMDL was observed in the meta-regression analysis. However, BMD calculation model, sex, and urinary cadmium concentration were not significantly associated with BMDL. Ethnic differences and age effect on BMDL could not be evaluated in the meta-regression, because the selected populations were mostly Asian populations and only limited number of studies reported the mean age of study participants. The cut-off point in the calculation of the BMD is its critical point. In an analysis by the European Food Safety Authority [9], the cut-off point was determined either statistically or biologically. Statistically, the cut-off point was defined as corresponding to the 95^th^ percentile of β2-MG at background exposure. The biological-based cut-offs for β2-MG were 300 and 1000 μg/g creatinine. A β2-MG level greater than 300 μg/g creatinine is considered adverse, and a level greater than 1000 μg/g creatinine is considered irreversible [31,32]. Nakagawa et al. [33] conducted a prospective cohort study in which the mortality rate was higher in the group with 300 to 1000 μg/g creatinine compared to the group with <300 μg/g creatinine after 9 years of follow up. In the studies selected for the present meta-analysis, a wide range of cut-off points have been used, and all the studies determined the cut-off point statistically. Lower background probability of adverse response yields higher BMD due to the s-shaped dose-response curve [26]. Therefore, higher cut-off point generally yields lower background probability, resulting in a higher BMD. The hybrid approach was applied in several studies for β2-MG [27–29]. The hybrid approach allows for calculation of BMDL directly from continuous data, while in the conventional quantal analysis method, continuous data are dichotomized for analysis into quantal data according to cut-off point dividing the population into the affected and unaffected. However, the BMDs from hybrid approach, which fixed a 5% of background probability, were also significantly associated with cut-off point in the present meta-regression analysis. Thus careful consideration should be made to select cut-off point which denotes an abnormal level.

The excretion of urinary β2-MG and NAG, which are often used in cadmium toxicity studies, reflects tubular damage, whereas ALB reflects glomerular damage that is considered to be irreversible [1,34]. Thus, β2-MG and NAG are believed to be early markers of tubular dysfunction, and it has been suggested that NAG is a more sensitive marker for urinary cadmium than β2-MG [35–37]. This led us to conduct an analysis to determine whether there were differences between NAG and β2-MG in response to the different levels of urinary cadmium concentration examined in the present study. Meta-analyses were performed by dividing the data into two groups based on urinary concentration. It was hypothesized that NAG showed smaller BMDL values in the lower concentration group (< 2 μg/g creatinine). However, there was not a large difference between β2-MG and NAG for both the low and high cadmium concentration groups, although the cut-off point of 2-MG and UNAG are different in those studies, which might significantly influence the BMDL values.

## Conclusion

The BMD for cadmium was not significantly different between hybrid and conventional quantal data approaches, but was significantly associated with the cut-off point defining the abnormal level of renal dysfunction markers.

## Supporting Information

S1 Fig PRISMA 2009 Checklist. (PDF)
